# State-Dependent Missingness in Hidden Markov Models, with an Application to Drop-Out in a Clinical Trial

**DOI:** 10.1017/psy.2024.3

**Published:** 2025-01-03

**Authors:** Maarten Speekenbrink, Ingmar Visser

**Affiliations:** 1 Department of Experimental Psychology, University College London, London, UK; 2 Department of Psychology, University of Amsterdam, Amsterdam, Netherlands

**Keywords:** hidden Markov models, longitudinal data, missing data, missing not at random

## Abstract

Establishing the effectiveness of treatments for psychopathology requires accurate models of its progression over time and the factors that impact it. Longitudinal data is however fraught with missingness, hindering accurate modeling. We re-analyse data on schizophrenia severity in a clinical trial using hidden Markov models (HMMs). We consider missing data in HMMs with a focus on situations where data is missing not at random (MNAR) and missingness depends on the latent states, allowing symptom severity to indirectly impact probability of missingness. In simulations, we show that including a submodel for state-dependent missingness reduces bias when data is MNAR and state-dependent, whilst not reducing accuracy when data is missing-at-random (MAR). When missingness depends on time, a model that allows missingness to be both state- and time-dependent is unbiased. Overall, these results show that modelling missingness as state-dependent and including relevant covariates is a useful strategy in applications of HMMs to time-series with missing data. Applying the model to data from a clinical trial, we find that drop-out is more likely for patients with less severe symptoms, which may lead to a biased assessment of treatment effectiveness.

## 1 Introduction

The progression of mental health and the search for effective interventions to improve it naturally lead to longitudinal data. Determining the impact of personality and other factors on the progression of a disorder is vital to understanding mental health dynamics and the effectiveness of treatments. Longitudinal data are unfortunately fraught with missingness, due to complete or partial drop-out of patients. Such missingness can severely affect the validity of inferences from the data. For example, if patients who react adversely to medication drop out of the study, this may lead to an unwarranted favourable evaluation of the effectiveness of the medication, as the results do not take into consideration patients who actually were worse off after taking the medication. It is therefore vital to properly address missing data in such studies.

The progression of disease and mental health is increasingly studied using hidden Markov models. Hidden Markov models (Rabiner, 1989; Visser & Speekenbrink, 2022) are suitable for categorical or metric time-series and longitudinal data governed by an underlying discrete process. In the context of longitudinal data, these models are also known as latent Markov models (Bartolucci et al., 2012). In these models, health status is considered a discrete state from a finite set, rather than a continuous variable. The focus is on how patients transition between healthy and less healthy states, either naturally or as a consequence of interventions. For example, Hosenfeld et al. (2015) studied patients transitioning in and out of major depressive episodes. Other recent applications have focused on patients with diagnosed depression (Catarino et al., 2020), bipolar disorder (Prisciandaro et al., 2019), and schizophrenia (Boeker et al., 2021). These applications of hidden Markov models are usually limited to complete data or otherwise ignore reasons for missing data. Here, we consider data from a randomized control trial testing the effectiveness of medication in the treatment of schizophrenia. As in many longitudinal studies, there is substantial missing data in this study. The aim of the present paper is to show how this missingness can be meaningfully addressed in applications of hidden Markov models to clinical studies and other data, by allowing missingness to depend on the underlying latent health state as well as other variables such as measurement occasion and intervention status.

There is relatively little work on dealing with missing data in hidden Markov models. Albert (2000), Deltour et al. (1999), and Yeh et al. (2010) consider missing data in Markov chains with observed states. Paroli and Spezia (2002) consider calculation of the likelihood of a Gaussian hidden Markov model when observations are missing at random (MAR). Yeh et al. (2012) discuss the impact of ignoring missingness when missing data is, and is not, ignorable. They show that if missingness depends on the hidden states, i.e., missingness is state-dependent, this results in biased parameter estimates when this missingness is ignored. However, they offer no solution to this problem. The objective of this paper is to do so. Our approach is related to the work of Yu and Kobayashi (2003), who allowed for state-dependent missingness in a hidden semi-Markov model with discrete (categorical) outcomes. Following Bahl et al. (1983), their solution is to code missingness into a special “null value” of the observed variable, effectively making the variable fully observed. Here, we instead model missingness with an additional (fully observed) indicator variable. This, we believe, is conceptually simpler, and makes it straightforward to add additional covariates to model the probability of missing values. This approach is also taken by Bartolucci and Farcomeni (2015), who restrict their model to the case of dropout in longitudinal data (where data is complete up to the point of dropout, after which all data is missing) rather than missing data more generally (where data can be missing at any time point).

The remainder of this paper is organized as follows: We start with an overview of the data measuring the severity of schizophrenic symptoms in a clinical trial (Hedeker & Gibbons, 1997) and a brief discussion about the usefulness of applying hidden Markov models to this type of data. This is followed by a brief overview of hidden Markov models and the definition of ignorable and non-ignorable missing data as established by Rubin (1976) and Little and Rubin (2014). We then consider both types of missing data in the context of hidden Markov models, and address the case of state-dependent missingness. We then present an inhomogeneous hidden Markov model for longitudinal data with state-dependent missingness and detail its estimation via expectation-maximization. In a series of simulation studies, we show how including a submodel for state-dependent missingness provides better estimates of the model parameters when missingness is state-dependent. When data is in fact MAR, the model with state-dependent missingness is not fundamentally biased, although care must be taken to include relevant covariates, such as e.g., time. These models are then applied to the dataset on the severity of schizophrenic symptoms in a clinical trial (Hedeker & Gibbons, 1997). We end by discussing the implications of this modeling exercise.

### 1.1 The National Institute of Mental Health Schizophrenia Collaborative Study

The National Institute of Mental Health Schizophrenia Collaborative Study assesses treatment-related changes in overall severity of schizophrenia. In the study, 437 patients diagnosed with schizophrenia were randomly assigned to receive either a placebo (108 patients) or one of three different anti-psychotic drugs (329 patients). The severity of their illness was rated by a clinician at baseline (week 0), and at subsequent one week intervals (weeks 1–6), with week 1, 3, and 6 as the intended main follow-up measurements. Measurements on the non-main measurement weeks (week 2, 4, and 5) are overwhelmingly missing with some patients having measurements in these weeks instead of the main measurement weeks. This data has been made publicly available by Don Hedeker^1^ and has been analyzed numerous times. In particular, Hedeker and Gibbons (1997) focused on pattern mixture methods to deal with missing data. Yeh et al. (2010) and Yeh et al. (2012) applied Markov and hidden Markov models, respectively, assuming ratings were MAR.

**Figure 1:**
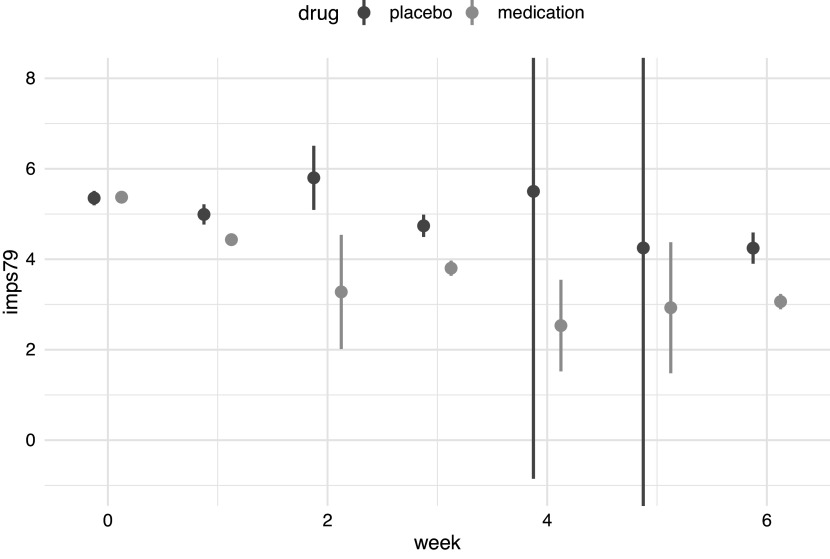
Average ratings of the severity of illness (IMPS item 79) by week and drug type. Bars depict 95% confidence intervals. Note that for the placebo group, confidence intervals at week 4 and 5 extend beyond the plot due to the small number of observations.

Our analysis focuses on a single item of the Inpatient Multidimensional Psychiatric Scale (Lorr & Klett, 1966), which rates illness severity on a scale from 1 (“normal”) to 7 (“among the most extremely ill”).^2^ The average severity ratings at each week are shown in Figure 1. As can be seen there, ratings at week 6 appear lower than those in week 0, especially for patients receiving medication. At week 6, patients who received the placebo had more severe illness than those receiving medication, with a difference in mean IMPS score of 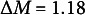 , 95% CI 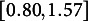 , 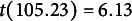 , 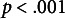 . There is however substantial missing data. Most participants were measured on week 0 (99.31%) and 1 (97.48%), whilst the other main measurement points at week 3 (85.58%) and 6 (76.66%) show more missing values. For a few participants, ratings were instead obtained on week 2 (3.2%), 4 (2.52%), and/or 5 (2.06%). Even when ignoring these rare deviations from the main measurement points, there is a clear potential issue with missing data and attrition, with 75.29% being measured the intended four times or more, and 15.1% rated on just three occasions, and 9.61% only twice.

Further insight into the extent of the missingness can be gained by studying the attrition or drop-out rates and the occurrence of intermittent missingness. First, 312 out of 437 patients have measurements at all four main measurement occasions. Second, 3 patients dropped out after measurement occasion 1, 45 patients after 2, and 53 patients after 3 measurement occasions. This leaves 24 patients with intermittent missingness patterns. In particular, 13, 5, and 3 patients have missing data at main measurement occasions 1, 2, or 3 respectively. Finally, 2 patients had missing data at main measurement occasions 2 and 3, and 1 patient had missing data at main measurement occasions 2 and 4.

**Figure 2:**
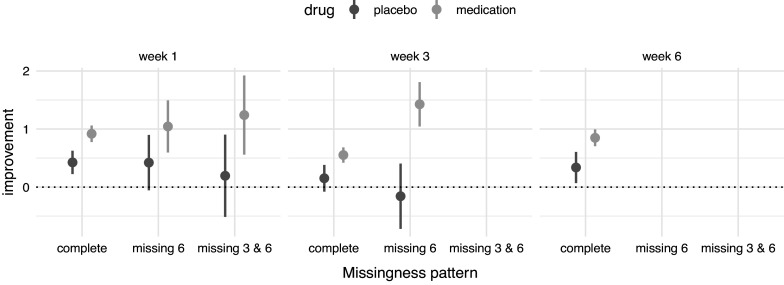
Improvement in symptoms at the main measurement weeks by missingness pattern and drug status. Note that missingness pattern concerns solely the main measurement weeks. Improvement are differences in scores between the main measurement weeks. Dots represent means and ranges 95% confidence intervals.

Focusing on the four main measurement weeks, Figure 2 compares the improvement in illness from week 0 of patients with missing data in both week 3 and week 6, patients with only missing data in week 6, and patients with complete data. This figure shows some clear differences between patients with and those without missing data. Differences are particularly evident at week 3, where patients in the medication group with missing data in week 6 have improved more than patients in the medication group without missing data. Drop-out of patients who respond well to medication may bias the assessment of treatment effectiveness, such that the treatment is deemed less effective than it is in reality. The question is then whether drop-out is random, or related to treatment effectiveness and/or illness severity. Modeling of these data should answer the question whether the origin of these differences in improvement is an actual difference in treatment effectiveness or an artifact caused by missingness.

To gain initial insight into patterns underlying the missing data, we modeled whether the IMPS rating was missing or not with a logistic regression model. Predictors in the model were a dummy-coded variable drug (0 for placebo, 1 for medication), week (from 0 to 6) as a metric predictor, and a dummy-coded variable main (1 for main measurement occasion, 0 otherwise) to indicate whether the rating was at a main measurement occasion (i.e., at week 0, 1, 3, or 6). We also included an interaction between drug and week, and between drug and main. The results of this analysis (Table 1) show a positive effect of week (such that missingness increases over time), and a negative effect of main, with (many) more missing values on weeks which are *not* the main measurement occasions. The positive effect of week is a clear sign of attrition. A remaining question is whether this attrition is related to the severity of the illness, in which case the ratings at week 6 would provide a biased view on the true severity of illness after six weeks of treatment with a placebo or medicine. There are different methods to address this, and many have been already applied to this particular dataset. For example, Hedeker and Gibbons (1997) used a pattern mixture approach with linear mixed-effects models and showed that improvement depends both on the type of drug and whether patients drop-out or not. Here, we suggest an alternative approach, incorporating a model of missingness into a hidden Markov model, thereby allowing missingness to depend on the latent state as well as observable features such as the measurement week.

**Table 1:** Results of a logistic regression analysis modeling missingness as a function of drug, week, and whether the week was a main measurement occasion or not

	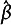	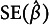	*z*	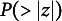
(Intercept)	1.921	0.393	4.884	0.000
Drug	0.433	0.463	0.936	0.349
Week	0.496	0.068	7.353	0.000
Main	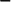 5.381	0.382	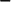 14.103	0.000
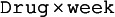	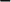 0.112	0.081	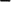 1.395	0.163
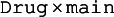	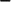 0.596	0.446	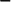 1.335	0.182

### 1.2 Hidden Markov models (HMM)

Let 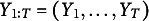 denote a time series of *D*-variate observations 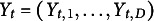 , and let 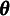 denote a vector of model parameters. A HMM associates observations with a time series of hidden (or latent) discrete states 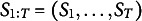 . In a first-order HMM, it is assumed that each state 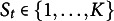 depends only on the immediately preceding state 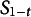 , and that, conditional upon the hidden states, the observations 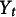 are independent: (1)

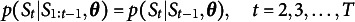


(2)

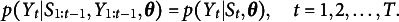

With these conditional independencies, the joint distribution of observations and states can be factored as (3)

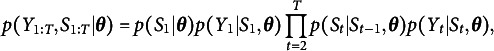

where 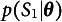 is the initial state distribution at time 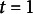 . The likelihood function (i.e., the marginal distribution of the observations as a function of the model parameters) can then be written as (4)

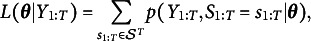

where the summation is over all possible state sequences (i.e., 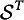 is the set of all possible sequences of states). Rather than actually summing over all possible state sequences, the forward-backward algorithm (Rabiner, 1989) is used to efficiently calculate this likelihood. For more information on HMMs, see also Visser and Speekenbrink (2022).

### 1.3 Missing data

The canonical references for statistical inference with missing data are Rubin (1976) and Little and Rubin (2014). Here we summarize the main ideas and results from those sources, as relevant to the present topic. For ease of presentation, we consider the case of a single *D*-variate time-series 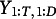 here.

Let 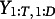 , the sequence of all *D*-variate response variables, be partitioned into a set of observed values, 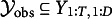 , and a set of missing values, 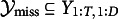 , with 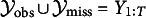 and 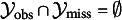 . Let 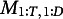 be a matrix of indicator variables with values 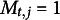 if 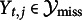 (the observation of dimension *j* at time *t* is missing), and 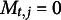 otherwise.

In addition to 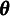 , the parameters of the hidden Markov model for the observed data *Y*, let 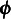 denote the parameter vector of the statistical model of missingness (i.e., the model of 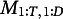 ). We can define the “full” likelihood function as (5)

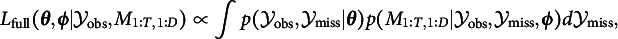

that is, as any function proportional to 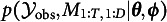 . Note that this is a marginal density, hence the integration over all possible values of the missing data. In this general case, we allow missingness to depend on the “complete” data 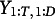 , so including the missing values 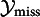 (for instance, it might be the case that missing values occur when the true value of 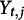 is relatively high).

The likelihood for the observed data, ignoring the missing values, can be defined as (6)

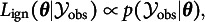

that is, as any function proportional to 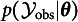 . An important question is when inference for 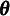 based on (5) and (6) give the same results. Note that both likelihood functions need only be known up to a constant of proportionality as only relative likelihoods need to be known for maximizing the likelihood or computing likelihood ratio’s. The question is thus when (6) is proportional to (5).

As shown by Rubin (1976), inference on 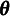 based on (5) and (6) will give identical results when (1) 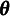 and 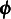 are separable (i.e., the joint parameter space is the product of the parameter space for 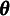 and 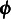 ), and (2) the following holds: (7)

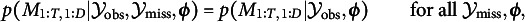

i.e., whether data is missing does not depend on the missing values. In this case, data is said to be missing at random (MAR), and the joint density can be factored as 

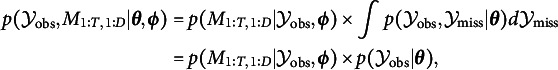

which indicates that, as a function of 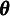 , 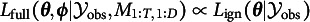 . Hence, when data is MAR, the missing data, and the mechanism leading to it, can be ignored in inference of 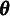 . A special case of MAR is data which is “missing completely at random” (MCAR), where (8)

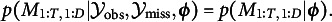



When the equality in (7) does not hold, data is said to be missing not at random (MNAR). In this case, ignoring the missing data will generally lead to biased parameter estimates of 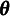 . Valid inference of 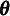 requires working with the full likelihood function of (5), so explicitly accounting for missingness.

### 1.4 Missing data in HMM

A HMM by definition includes missing data, as the hidden states *S* are unobservable (i.e., always missing). When there are no missing values for the *D*-dimensional response variable 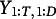 , it is straightforward to show that inference on 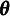 in HMMs targets the correct likelihood. Let 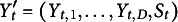 define a 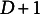 -dimensional variable, for which 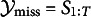 and 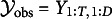 . Then 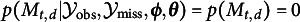 , for all 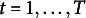 , 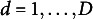 , and 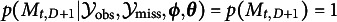 , for all 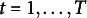 . Therefore, the missing states can be considered MCAR.

As the hidden states in a HMM are MCAR, we will ignore them in the missingness models in the remainder, so that 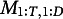 corresponds solely to the missing values for the observable variables. We will now focus on the case where the observable response variables 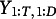 do have missing values. The full likelihood, which involves marginalizing over the hidden states, can be defined as (9)

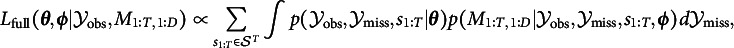

while the likelihood ignoring missing values can be defined as (10)

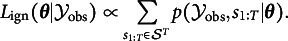



#### 1.4.1 MAR

When the data is MAR (7), then (11)

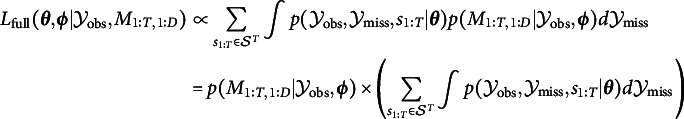

and hence missingness is ignorable in inference of 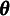 . Assuming that the *D*-variate responses are conditionally independent: (12)

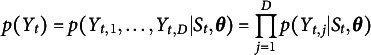

and defining (13)

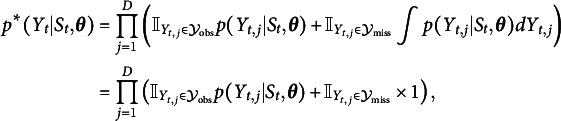

where the indicator variable 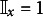 if condition *x* is true and 0 otherwise, we can write the part of the full likelihood (11) relevant to inference on 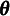 as 

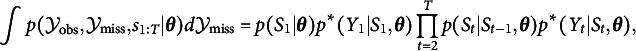

which shows that a principled way to deal with missing observations is to set 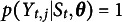 for all 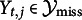 . Note that it is necessary to include time points with missing observations in this way to allow the state probabilities to be computed properly. While this result is known (e.g., Zucchini et al., 2017), we have not come across its derivation in the form above.

#### 1.4.2 State-dependent missingness (MNAR)

If data is not MAR, there is some dependence between whether observations are missing or not, and the true unobserved values. There are many forms this dependence can take, and modeling the dependence accurately may require substantial knowledge of the domain to which the data applies. Here, we take a pragmatic approach, and model this dependence via the hidden states. We assume *M* and *Y* are conditionally independent, given the hidden states: 

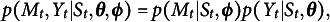

where 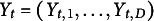 and hence 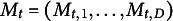 can be multivariate. Conditional independence between responses and missingness is not an overly restrictive assumption, as the number of hidden states can be chosen to allow for intricate patterns of (marginal) dependence between *M* and *Y* at a single time point, as well as over time. For example, increased probability of missingness for high values of *Y* can be captured through a state which is simultaneously associated with high values of *Y* and a high probability of 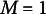 . A high probability of a missing observation at 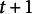 *after* a high (observed) value of 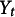 can be captured with a state *s* associated with high values of *Y*, a state 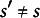 associated with a high probability of 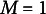 , and a high transition probability 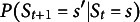 between these states.

Under the assumption that missingness depends solely on the hidden states, such that 

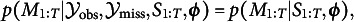

the full likelihood can be stated as 

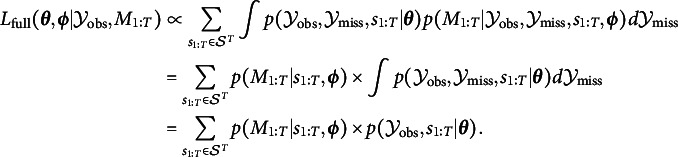

This shows that, although *M* does not directly depend on 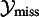 , because both *M* and *Y* depend on the state *S*, the role of the 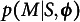 term is more than a scaling factor in the likelihood, and hence missingness is not ignorable.

### 1.5 Overview

When data is MNAR and missingness is not ignorable, valid inference on 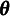 requires including a submodel for *M* in the overall model. That is, the HMM should be defined for both *Y* and *M*. The objective of the present paper is to show the potential benefits of including a relatively simple model for *M* in HMMs, by assuming missingness is state-dependent. We first provide results from a simulation study. The simulations assess the accuracy of parameter estimates and state recovery in situations where missingness is MAR or MNAR and dependent on the hidden state, in situations where the state-conditional distributions of the observations are relatively well separated or more overlapping. We also discuss a situation where missingness depends on the true value of *Y*, and one where missingness is time-dependent (but not state-dependent). The latter is a situation where missingness is in fact MCAR, and where a misspecified model which assumes missingness is state-dependent might lead to biased results. Finally, we apply the models to the real data from a clinical trial comparing the effect of real and placebo medication on the severity of schizophrenic symptoms.

## 2 Inhomogeneous HMM for multivariate longitudinal data with state-dependent missingness

In the remainder, we will consider HMMs with *K* states for longitudinal data consisting of sets of time-series (e.g., time-series for different patients) which may differ in length. Let 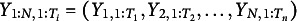 denote such a set of *N* time-series 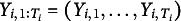 , each of length 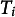 . Whilst we will focus on univariate responses in the remainder, the results apply directly to *D*-variate responses, 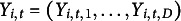 , as long as conditional independence (12) holds. We will allow state-transitions to be inhomogeneous (i.e., time-variant) by including covariates 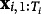 on the initial state probabilities and state-transition probabilities. Here, we use multinomial logistic regressions: 

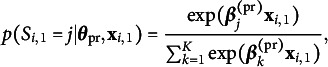

and 

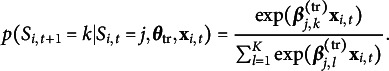

Note that for identification, 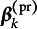 and 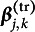 should be fixed to 0 for one state 
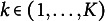
 , and that usually, 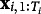 will include a constant term for the intercept.

We allow responses 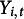 to depend on hidden states 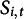 and covariates 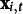 . For continuous-valued variates 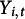 , we can for example use linear regressions: 

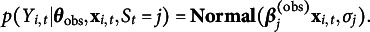

Finally, missingness 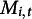 is allowed to depend on the hidden states and covariates. Here, we use logistic regression 

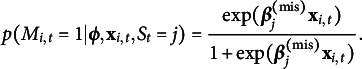



### 2.1 Estimation via expectation–maximization

Then the following, let 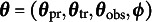 denote all the model parameters, with 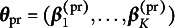 denoting the parameters for the initial state probabilities, 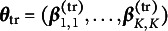 the parameters for the state-transition probabilities, 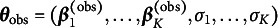 the parameters for the state-conditional observation densities, and 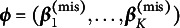 the parameters for the state-conditional missingness probabilities.

These parameters can be estimated through the expectation–maximization (EM) algorithm, which in the context of HMM is also known as the Baum–Welch algorithm. The EM algorithm consists of iteratively maximizing the expected joint log-likelihood 

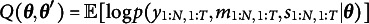

where the expectation is taken with respect to 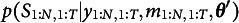 . Note that the expectation is based on initial parameter values 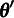 , whilst the joint log likelihood is defined over parameter values 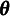 .

The expected joint log-likelihood can be written as (14)

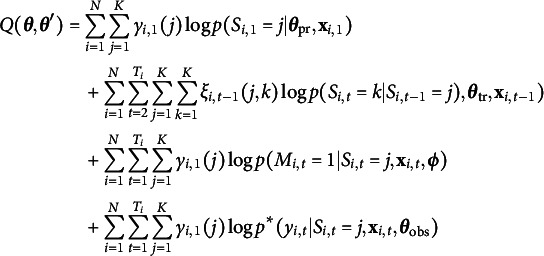

where 

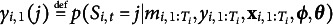

is the posterior probability of state 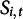 and 

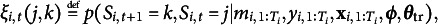

is the joint posterior probability of states 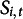 and 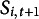 . These probabilities can be efficiently computed via the forward-backward algorithm (Rabiner, 1989). We define the forward-variable 

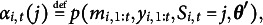

which can be computed iteratively as (15)

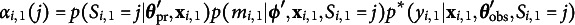

and for 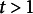 , as (16)

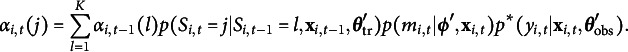

We also define the backward-variable 

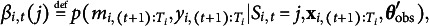

which is initialized at 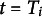 as (17)

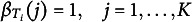

and then for each time 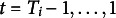 as (18)

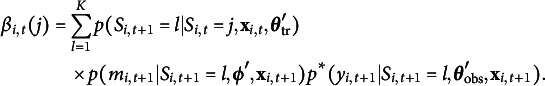

Using the forward and backward variables, we can compute the posterior state probabilities as (19)

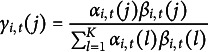

and (20)

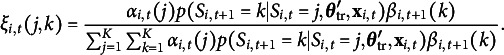



Note that the expected joint log-likelihood (14) is the sum of four weighted log-likelihoods, one for each set of parameters 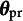 , 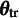 , 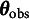 , and 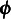 . Maximizing the expected joint log-likelihood therefore consists of separately maximizing four weighted likelihoods. When the initial states, state transitions, responses and missingness indicators are modeled with generalized linear models and multinomial logistic regression models, as we have done here, we can then rely on the standard maximum likelihood estimation procedures for these models (see McCullagh & Nelder, 1989), using the 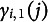 and 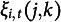 values as case-weights (see also Visser & Speekenbrink, 2022).

The full EM algorithm can be specified as

1. Start with initial parameters 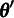 .
2. Do until convergence:
  - For 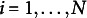 , 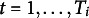 , 
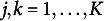
 , compute 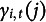 (19) and 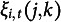 (20) via the forward-backward recursions (15, 16, 17, 18).
  - Obtain new estimates 

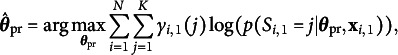




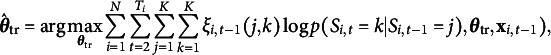




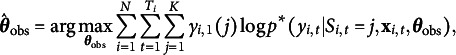

    and 

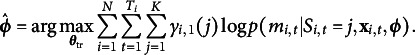

  - If 
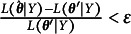
 (e.g., 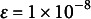 ), assume convergence.
  - Set 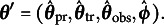

The EM algorithm is guaranteed to converge to a local maximum of the likelihood. Assessing whether the algorithm converged to the global maximum is not possible in general. To increase the chances to obtain the global maximum likelihood parameters, the algorithm can be run many times, each time using different starting values 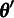 . Starting values for 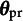 and 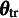 can be derived by assuming uniform distributions for 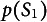 and 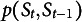 , or sampling these from suitable Dirichlet distributions. Starting values for 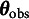 are less straightforward to choose in general, and arguably more important. One method is to randomly sample state probabilities 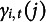 from a suitable Dirichlet distribution, and then set 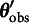 to the maximum likelihood estimates as in step b. This usually provides valid starting values and is the default option in depmixS4 (Visser & Speekenbrink, 2010).

### 2.2 Model selection, checking, and standard errors

An important consideration when using HMMs is the number of latent states *K*. This is generally determined by estimating models with different values for *K* and then choosing the best one via model selection criteria such as the Akaike information criterion (AIC; Akaike, 1998) and the Bayesian information criterion (BIC; Schwarz, 1978). For present purposes, another consideration is choosing between a MAR and MNAR model. As our MNAR model contains and additional missingness variable *M*, the AIC and BIC measures cannot be used directly, as the models target different likelihoods (one for the joint distribution of *Y* and *M*, and one for the distribution of just *Y*). A suitable alternative is to fix the probability of missingness in the MNAR model to be identical over the states, effectively creating a MAR model. As this MAR model is nested within the MNAR model, a general likelihood ratio test 

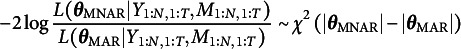

may be used to determine whether the MNAR model outperforms the MAR model ( 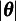 denotes the number of free parameters in 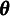 ).

Another consideration is whether the distributional assumptions for the state-conditional distributions 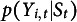 are reasonable. Zucchini et al. (2017) propose computing “pseudo-residuals” from the cumulative probabilities 

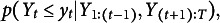

which are converted to the corresponding quantiles of a standard Normal distribution. If the model fits the data, then these quantiles will follow a standard Normal distribution. They show that the cumulative probability can be computed as a weighted sum 

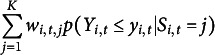

with 

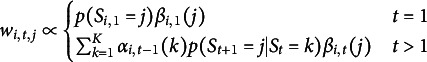

where the weights are normalized such that 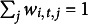 .

For inference on model parameters, standard errors and confidence intervals are of importance. There are several methods to compute (approximate) standard errors for maximum likelihood parameter estimates of HMM (Visser et al., 2000). The standard approach for obtaining standard errors from the Hessian matrix of second derivatives of the log-likelihood function is computationally tricky, as discussed in Visser et al. (2000), although Lystig and Hughes (2002) provide an elegant solution to overcome this computational challenge. Other methods include likelihood profiles and bootstrapping. Here we use a finite differences approach to estimate the Hessian matrix, which in turn is used to compute confidence intervals for the estimated parameters. Note that the method for finite differences proposed in Visser et al. (2000) was updated in Visser and Speekenbrink (2022) and implemented in depmixS4 (Visser & Speekenbrink, 2010). This updated finite difference method provides standard error estimates that are as accurate as those provided by bootstrapping methods (which are much more computationally expensive).

## 3 Simulation study

**Figure 3:**
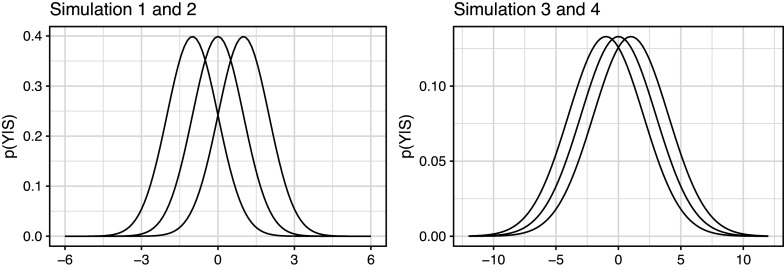
State-conditional response distributions in the simulation studies. In Simulation 1 and 2, states are reasonably well-separated, although there is still considerable overlap of the distributions. In Simulation 3 and 4, states are less well-separated.

To assess the potential benefits of including a state-dependent missingness model in a HMM, we conducted a simulation study, focusing on a three-state HMM with a univariate Normal distributed response variable^3^. We simulated four scenario’s. In Simulation 1 and 2, the states are reasonably well-separated with means 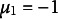 , 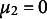 , 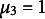 and standard deviations (SD) 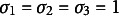 (see Figure 3). Note that there is still considerable overlap in the state-conditional response distributions, as would be expected in many real applications of HMMs. In Simulation 1, missingness was state-dependent (i.e., MNAR), with 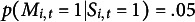 , 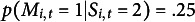 , and 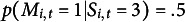 . In Simulation 2, missingness was independent of the state (MAR), with 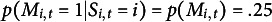 . In Simulation 3 and 4 (Figure 3), the states were less well-separated, with means as for Simulation 1 and 2, but SD 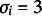 (see Figure 3). Here, the overlap of the state-conditional response distributions is much higher than in Simulation 1 and 2, and identification of the hidden states will be more difficult. In Simulation 3, missingness was state-dependent (MNAR) in the same manner as Simulation 1, while in Simulation 4, missingness was state-independent (MAR) as for Simulation 2. In all simulations, the initial state probabilities were 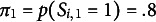 , 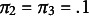 , and the state-transition matrix was

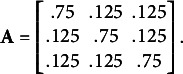

In each simulation, we simulated a total of 1000 data sets, each consisting of 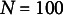 replications of a time-series of length 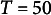 . We denote observations in such replicated time series as 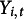 , with 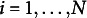 and 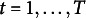 . Data was generated according to a 3-state hidden Markov model. For MAR cases, the non-missing observations are distributed as (21)

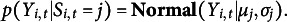

In the MNAR cases, the missingness variable *M* and the response variable *Y* were conditionally independent given the hidden state: (22)

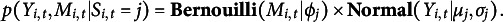

Data sets were simulated by first generating the hidden state sequences 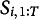 according to the initial state and transition probabilities. Then, the observations 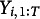 were sampled according to the state-conditional distributions 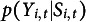 . Finally, random observations were set to missing values according to the missingness distributions 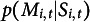 .

We fitted two 3-state HMM to each data-set. In the MAR models, observed responses were assumed to be distributed according to (21), and in the MNAR models, the observed responses and missingness indicators were assumed to be distributed according to (22). Parameters were estimated by maximum likelihood, using the EM algorithm, as implemented in depmixS4 (Visser & Speekenbrink, 2010). To speed up convergence, starting values were set to the true parameter values. Although such initialization is obviously not possible in real applications, we are interested in the quality of parameter estimates at the global maximum likelihood solution, and setting starting values to the true parameters makes it more likely to arrive at the global maximum. In real applications, one would need to use a sufficient number of randomly generated starting values to find the global maximum.

The results of Simulation 1 (Table 2) show that, when states are relatively well separated, both models provide parameter estimates which are, on average, reasonably close to the true values. Both models have the tendency to estimate the means as more dispersed, and the SD as slightly smaller, then they really are. While wrongly assuming MAR may not lead to overly biased estimates, we see that the MAE for the MNAR model is always smaller than that of the MAR model, reducing the estimation error to as much as 58%. Over all parameters, the relative MAE of the models is 0.77 on average, which shows a clear advantage of the MNAR model. As such, accounting for state-dependent missingness increases the accuracy of the parameter estimates. We next consider recovery of the hidden states, by comparing the true hidden state sequences to the maximum a posteriori (MAP) state sequences determined by the Viterbi algorithm (see Rabiner, 1989; Visser & Speekenbrink, 2022). The MAR model recovers 53.13% of the states, while the MNAR model recovers 62.86% of the states. The accuracy in recovering the hidden states is thus higher in the model which correctly accounts for state-dependent missingness. Whilst the performance of neither model may seem overly impressive, we should note that recovering the hidden states is a non-trivial task when the state-conditional response distributions have considerable overlap (see Figure 3) and states do not persist for long periods of time (here, the true self-transitions probabilities are 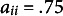 , meaning that states have an average run-length of 4 consecutive time-points). When ignoring time-dependencies and treating the observed data as coming from a bivariate mixture distribution over *Y* and *M*, the maximum accuracy in classification would be 50.09% for this data. The theoretical maximum classification accuracy for the hidden Markov model is more difficult to establish, but simulations show that the MNAR model with the true parameters recovers 66.51% of the true states. For the MAR model, the approximate maximum classification accuracy is 58.06%.

**Table 2:** Results of Simulation 1 (MNAR, low variance)

		MAR			MNAR			
Parameter	True value	Mean	SD	MAE	Mean	SD	MAE	rel. MAE
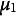	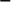 1.000	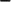 1.010	0.131	0.094	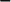 1.017	0.097	0.072	0.767
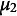	0.000	0.015	0.277	0.223	0.014	0.228	0.180	0.807
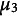	1.000	1.113	0.286	0.231	1.053	0.252	0.186	0.803
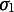	1.000	0.998	0.051	0.033	0.995	0.034	0.026	0.785
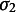	1.000	0.972	0.111	0.079	0.979	0.085	0.064	0.809
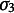	1.000	0.959	0.104	0.077	0.979	0.085	0.061	0.801
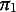	0.800	0.834	0.146	0.117	0.776	0.110	0.083	0.715
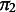	0.100	0.118	0.152	0.111	0.131	0.130	0.105	0.944
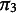	0.100	0.049	0.048	0.062	0.093	0.066	0.055	0.890
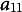	0.750	0.774	0.080	0.064	0.743	0.055	0.039	0.613
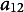	0.125	0.144	0.094	0.068	0.139	0.077	0.061	0.896
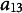	0.125	0.082	0.055	0.057	0.118	0.054	0.044	0.765
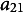	0.125	0.144	0.086	0.065	0.124	0.062	0.048	0.729
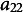	0.750	0.759	0.116	0.087	0.754	0.096	0.070	0.812
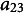	0.125	0.097	0.082	0.068	0.122	0.075	0.058	0.850
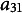	0.125	0.146	0.085	0.068	0.118	0.050	0.039	0.579
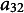	0.125	0.166	0.128	0.103	0.138	0.092	0.070	0.679
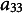	0.750	0.688	0.111	0.090	0.744	0.076	0.056	0.623
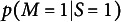	0.050	–	–	–	0.048	0.021	0.017	–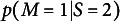	0.250	–	–	–	0.247	0.073	0.057	–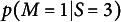	0.500	–	–	–	0.507	0.058	0.040	–

*Note*: Values shown are the true value of each parameter, and the mean (mean), standard deviation (SD), and mean absolute error (MAE) of the parameter estimates, for both the MAR and MNAR model. The value of “rel. MAE” is the ratio of the mean absolute error of the MNAR over the MAR model.

The results of Simulation 2 (Table 3) show that when data is in fact MAR, both models provide roughly equally accurate parameter estimates. Whilst the MNAR model does not provide better parameter estimates, including a model component for state-dependent missingness does not seem to bias parameter estimates compared to the MAR model. As can be seen, the state-wise missingness probabilities are, on average, close to the true values of .25. Over all parameters, the relative MAE of the models is 1.003 on average, which shows the models perform equally well. In terms of recovering the hidden states, the MAR model recovers 55.6% of the states, while the MNAR model recovers 55.63% of the states. The somewhat reduced recovery rate of the MNAR model compared to Simulation 1 is likely due to the fact that here, missingness provides no information about the identity of the hidden state. For comparison, the maximum classification accuracy is 42.91% for a mixture model, and approximately 60.45% for the HMM.

**Table 3:** Results of Simulation 2 (MAR, low variance)

		MAR			MNAR			
Parameter	True value	Mean	SD	MAE	Mean	SD	MAE	rel. MAE
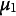	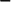 1.000	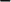 1.061	0.194	0.132	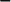 1.062	0.200	0.134	1.014
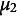	0.000	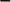 0.019	0.287	0.229	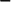 0.022	0.288	0.230	1.005
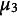	1.000	1.048	0.213	0.158	1.038	0.213	0.157	0.991
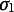	1.000	0.978	0.067	0.047	0.978	0.070	0.047	1.000
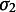	1.000	0.969	0.105	0.079	0.969	0.107	0.081	1.021
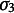	1.000	0.981	0.070	0.050	0.982	0.071	0.049	0.993
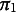	0.800	0.739	0.177	0.130	0.737	0.178	0.132	1.015
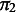	0.100	0.169	0.187	0.144	0.171	0.187	0.145	1.012
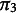	0.100	0.092	0.070	0.057	0.092	0.069	0.057	0.995
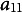	0.750	0.727	0.102	0.064	0.726	0.102	0.065	1.006
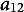	0.125	0.155	0.112	0.082	0.156	0.115	0.083	1.020
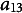	0.125	0.118	0.066	0.051	0.118	0.062	0.050	0.975
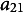	0.125	0.125	0.080	0.061	0.127	0.083	0.062	1.013
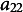	0.750	0.751	0.112	0.084	0.749	0.116	0.086	1.025
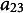	0.125	0.125	0.081	0.061	0.125	0.083	0.063	1.034
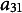	0.125	0.112	0.063	0.051	0.112	0.062	0.049	0.973
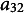	0.125	0.153	0.108	0.082	0.150	0.107	0.081	0.982
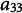	0.750	0.735	0.096	0.067	0.738	0.095	0.066	0.984
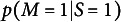	0.250	–	–	–	0.250	0.046	0.027	–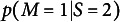	0.250	–	–	–	0.248	0.056	0.038	–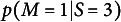	0.250	–	–	–	0.247	0.046	0.028	–

*Note*: Values shown are the true value of each parameter, and the mean (mean), standard deviation (SD), and mean absolute error (MAE) of the parameter estimates, for both the MAR and MNAR model. The value of “rel. MAE” is the ratio of the mean absolute error of the MNAR over the MAR model.

In Simulation 3 (Table 4) and 4 (Table 5) the states are less well-separated, making accurate parameter estimation more difficult. Here, the tendency to estimate the means as more dispersed and the SD as smaller than they are becomes more pronounced. For both models the estimation error in Simulation 3 (Table 4) is larger than for Simulation 1, but comparing the MAE for both models again shows the substantial benefits of including a state-dependent missingness model. Over all parameters, the relative MAE of the models is 0.658 on average, which shows the MNAR model clearly outperforms the MAR model. In terms of recovering the hidden states, the MAR model recovers 34.97% of the states, whilst the MNAR model recovers 45.27% of the states. As in Simulation 1, the MNAR model performs better in state identification. For both models, performance is lower than in Simulation 1, reflecting the increased difficulty due to increased overlap of the state-conditional response distributions (Figure 3). Indeed, the performance of the MAR model is close to chance (random assignment of states would give an expected accuracy of 33.33%). The maximum classification accuracy is 44.03% for a mixture model, and approximately 54.04% for the MNAR and 41.42% for the MAR HMM.

**Table 4:** Results of Simulation 3 (MNAR, high variance)

		MAR			MNAR			
Parameter	True value	Mean	SD	MAE	Mean	SD	MAE	rel. MAE
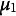	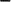 1.000	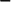 1.663	0.932	0.761	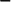 1.198	0.628	0.315	0.414
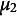	0.000	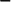 0.314	0.484	0.461	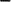 0.110	0.470	0.409	0.888
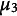	1.000	1.480	1.214	0.923	1.383	0.956	0.609	0.661
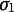	3.000	2.765	0.459	0.347	2.911	0.330	0.189	0.543
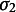	3.000	2.889	0.455	0.302	2.967	0.333	0.215	0.713
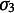	3.000	2.703	0.512	0.406	2.773	0.479	0.326	0.803
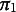	0.800	0.546	0.362	0.355	0.657	0.281	0.217	0.611
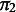	0.100	0.346	0.380	0.333	0.253	0.291	0.231	0.694
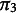	0.100	0.108	0.174	0.129	0.090	0.091	0.077	0.601
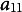	0.750	0.651	0.231	0.183	0.712	0.153	0.099	0.543
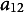	0.125	0.190	0.215	0.160	0.144	0.145	0.105	0.660
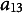	0.125	0.159	0.186	0.139	0.144	0.124	0.091	0.659
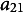	0.125	0.106	0.172	0.124	0.106	0.108	0.085	0.687
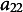	0.750	0.787	0.232	0.185	0.784	0.135	0.109	0.590
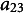	0.125	0.107	0.158	0.115	0.110	0.105	0.085	0.742
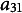	0.125	0.152	0.183	0.136	0.131	0.126	0.096	0.704
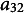	0.125	0.166	0.199	0.143	0.145	0.141	0.105	0.738
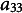	0.750	0.682	0.234	0.184	0.724	0.151	0.108	0.587
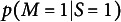	0.050	-	-	-	0.076	0.122	0.059	-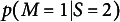	0.250	-	-	-	0.241	0.155	0.126	-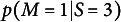	0.500	-	-	-	0.489	0.134	0.092	-

*Note*: Values shown are the true value of each parameter, and the mean (mean), standard deviation (SD), and mean absolute error (MAE) of the parameter estimates, for both the MAR and MNAR model. The value of “rel. MAE” is the ratio of the mean absolute error of the MNAR over the MAR model.

**Table 5:** Results of Simulation 4 (MAR, high variance)

		MAR			MNAR			
Parameter	True value	Mean	SD	MAE	Mean	SD	MAE	rel. MAE
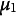	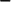 1.000	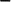 1.650	1.002	0.801	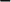 1.658	1.107	0.815	1.018
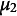	0.000	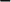 0.171	0.539	0.432	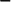 0.178	0.542	0.437	1.010
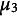	1.000	1.468	1.063	0.778	1.473	1.070	0.788	1.014
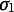	3.000	2.719	0.468	0.383	2.720	0.473	0.375	0.981
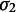	3.000	2.911	0.441	0.299	2.918	0.412	0.279	0.934
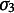	3.000	2.728	0.504	0.377	2.732	0.478	0.365	0.968
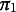	0.800	0.528	0.345	0.352	0.522	0.338	0.357	1.012
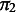	0.100	0.330	0.367	0.316	0.344	0.359	0.320	1.014
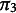	0.100	0.141	0.199	0.149	0.134	0.192	0.142	0.951
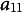	0.750	0.638	0.230	0.183	0.645	0.220	0.177	0.968
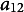	0.125	0.188	0.212	0.155	0.182	0.211	0.155	0.998
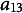	0.125	0.174	0.188	0.139	0.174	0.178	0.134	0.963
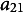	0.125	0.111	0.166	0.121	0.103	0.157	0.119	0.984
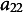	0.750	0.774	0.223	0.175	0.787	0.209	0.170	0.972
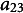	0.125	0.114	0.152	0.111	0.110	0.139	0.110	0.986
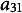	0.125	0.133	0.169	0.125	0.137	0.167	0.124	0.997
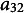	0.125	0.167	0.193	0.138	0.162	0.186	0.139	1.007
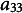	0.750	0.700	0.232	0.177	0.701	0.224	0.176	0.992
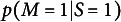	0.250	–	–	–	0.253	0.122	0.080	–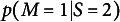	0.250	–	–	–	0.237	0.086	0.056	–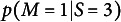	0.250	–	–	–	0.257	0.130	0.082	–

*Note*: Values shown are the true value of each parameter, and the mean (mean), standard deviation (SD), and mean absolute error (MAE) of the parameter estimates, for both the MAR and MNAR model. The value of “rel. MAE” is the ratio of the mean absolute error of the MNAR over the MAR model.

When missingness is ignorable (Simulation 4), like in Simulation 2, inclusion of a state-dependent missingness component in the HMM does not increase bias in parameter estimates. Over all parameters, the relative MAE of the models is 0.987 on average, which shows the models perform roughly equally well. The MAR model recovers 35.51% of the states, whilst the MNAR model recovers 35.5% of the states. For comparison, the maximum accuracy is 36.64% for a mixture model, and 42.51% for the HMM.

Taken together, these simulation results show that if missingness is state-dependent, there is a substantial benefit to including a (relatively simple) model for missingness in the HMM. When missingness is in fact ignorable, including a missingness model is superfluous, but does not bias the results. Hence, there appears to be little risk associated with including a missingness submodel in the HMM.

Four additional simulations were conducted to assess to what extent these results depend on the persistence of states and the homogeneity of state transition probabilities over the states. In these simulations, we used the relatively well-separated states of Simulations 1 and 2. In Simulation 5 and 6, we changed the initial state probabilities to a uniform distribution 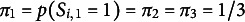 and the state-transition matrix to 

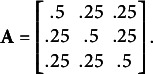

Thus, in these simulations, initial state identification may be more difficult, and the states are (even) less persistent than in Simulations 1 and 2. Full results are provided in the Appendix. When data is MNAR (Table A1), we again find a clear advantage of the MNAR model, with an average relative MAE of 0.886. The MAR model recovers 44.38% of the states, while the MNAR model recovers 51.62% of the states. When data is MAR (Table A2), both models perform roughly equally well, with an average relative MAE of 0.971. The MAR model recovers 46.33% of the states, while the MNAR model recovers 46.34% of the states. In Simulations 7 and 8, we used the same initial state probabilities as in Simulation 1 and 2, but changed the state-transition matrix to 

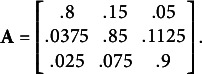

As such, the states persist longer than in Simulation 1 and 2, and persistence is furthermore dependent on the state. When data is MNAR (Table A3), we again find a clear advantage of the MNAR model, with an average relative MAE of 0.727. The MAR model recovers 64.52% of the states, while the MNAR model recovers 73.56% of the states. When data is MAR (Table A4), both models perform roughly equally well, with an average relative MAE of 1.003. The MAR model recovers 68.09% of the states, while the MNAR model recovers 68.03% of the states.

In a further simulation, we consider a more traditional case of MNAR data, where missingness depends on the underlying value of the response variable. More specifically, we model the probability of missingness as a function of the true value of the response 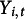 via a logistic regression: 

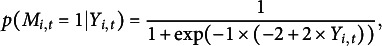

keeping the other parameters the same as in Simulation 1. Whilst missingness does not directly depend on the hidden state, because the true response values do depend on the states, the probability of missingness differs between the states, with approximately 6.8%, 22.5%, and 50% expected missing values in states 1, 2, and 3 respectively. As such, although the relation between the underlying true value of the response and missingness is not part of the MNAR model, we would expect the model to indicate state-dependent missingness. The results of this simulation (Table 6) show a clear bias in estimating the state-dependent means and SD: because the higher the value of the response, the higher the probability that value is missing, both state dependent means and SD are underestimated, particularly for state 3 where the probability of missingness is highest. Whilst bias in parameter estimates is evident in both the MAR and MNAR model, the latter performs better on average: over all parameters, the relative MAE of the models is 0.835 on average, which shows a clear advantage of the MNAR model. State recovery seems relatively unaffected by the bias in parameter estimates. The MAR model recovers 49.6% of the states, and the MNAR model recovers 59.15% of the states. These results are close to those of Simulation 1. Thus, for this more traditional form of MNAR data, the (misspecified) MNAR model again outperforms the MAR model, and state recovery seems mostly unaffected by the unavoidable bias in parameter estimates.

**Table 6:** Results of Simulation 9 (MNAR, related to true value)

		MAR			MNAR			
Parameter	True value	Mean	SD	MAE	Mean	SD	MAE	rel. MAE
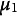	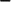 1.000	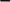 1.101	0.171	0.120	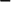 1.115	0.130	0.123	1.026
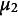	0.000	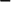 0.256	0.247	0.281	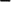 0.245	0.210	0.265	0.944
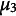	1.000	0.538	0.224	0.473	0.506	0.192	0.499	1.054
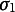	1.000	0.965	0.051	0.044	0.957	0.043	0.047	1.069
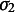	1.000	0.807	0.090	0.193	0.804	0.071	0.196	1.014
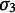	1.000	0.681	0.113	0.321	0.700	0.102	0.302	0.939
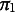	0.800	0.836	0.166	0.134	0.777	0.144	0.103	0.770
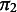	0.100	0.126	0.169	0.122	0.141	0.155	0.118	0.968
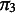	0.100	0.038	0.049	0.072	0.082	0.064	0.055	0.764
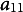	0.750	0.760	0.103	0.074	0.728	0.078	0.052	0.708
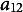	0.125	0.174	0.107	0.083	0.166	0.089	0.072	0.876
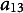	0.125	0.067	0.059	0.073	0.106	0.061	0.051	0.702
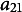	0.125	0.178	0.109	0.089	0.156	0.079	0.062	0.695
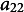	0.750	0.718	0.147	0.105	0.716	0.115	0.086	0.820
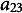	0.125	0.105	0.099	0.079	0.128	0.087	0.066	0.830
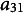	0.125	0.140	0.117	0.086	0.119	0.074	0.053	0.619
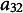	0.125	0.209	0.167	0.142	0.167	0.114	0.092	0.645
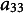	0.750	0.651	0.142	0.120	0.715	0.098	0.071	0.593
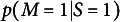	0.068	-	-	-	0.070	0.040	0.022	-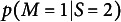	0.225	-	-	-	0.235	0.090	0.071	-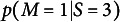	0.500	-	-	-	0.525	0.075	0.053	-

*Note*: Values shown are the true value of each parameter, and the mean (mean), standard deviation (SD), and mean absolute error (MAE) of the parameter estimates, for both the MAR and MNAR model. The value of “rel. MAE” is the ratio of the mean absolute error of the MNAR over the MAR model.

In a final simulation, we assessed the performance of the models when missingness is time-dependent, rather than state-dependent. Attrition is common in longitudinal studies, meaning that the probability of missingness often increases with time. In this simulation, the probability of missingness as a function of time *t* was modeled through a logistic regression model: (23)

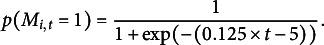

Here, the probability of missing data is very small (0.008) at time 1, but increases substantially to (0.777) at time 50. The other parameters were the same as in Simulation 1 and 2. In a model that specifies missingness as state-dependent, but not time-dependent, this could potentially result in biased parameter estimates. For instance, the increased probability of missingness over time may be accounted for by estimating states to have a different probability of missingness, and estimating prior and transition probabilities to allow states with a higher probability of missingness to occur more frequently later in time. In addition to the two HMMs estimated before, we also estimated a HMM with a state- and time-dependent model for missingness: (24)

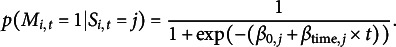

This model should be able to capture the true pattern of missingness, whilst the MNAR model which only includes state-dependent missingness would not.

The results (Table 7) show that, compared to the MAR model, the MNAR model which misspecifies missingness as state-dependent is inferior, resulting in more biased parameter estimates. Over all parameters, the relative MAE of these two models is 1.353 on average, indicating the MAR model outperforms the MNAR (state) model. To account for the increase in missing values over time, the MNAR (state) model estimates the probability of missingness as highest for state 2, which is estimated to have a mean of close to 0, but an increased SD to incorporate observations from the other two states. To make state 2 more prevalent over time, transition probabilities to state 2 are relatively low from state 1 and 3 (parameters 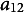 and 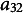 respectively), whilst self-transitions ( 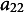 ) are close to 1 (meaning that once in state 2, the hidden state sequence is very likely to remain in that state). The prevalence of state 2 is thus increasing over time, and as this state has a higher probability of missingness, so is the prevalence of missing values. The MNAR (time) model, which allows missingness to depend on both the hidden states and time, performs only slightly worse than the MAR model, with an average relative MAE over all parameters of this model compared to the MAR of 1.042. However, the MNAR (time) model is able to capture the pattern of attrition (increased missing data over time), whilst the MAR model is not. As such, the MNAR (time) model may be deemed preferable to the MAR model, insofar as one is interested in more than modeling the responses *Y*. In terms of recovering the hidden states, the MAR model recovers 55.67% of the states, and the MNAR (time) model recovers 55.42% of the states. The misspecified MNAR (state) model recovers 50% of the states. The maximum classification accuracy for this data is 42.95% for a mixture model, and approximately 59.91% for the HMM.

**Table 7:** Results of Simulation 10 (time-dependent missingness, low variance)

		MAR			MNAR (state)			MNAR (time)				
Parameter	True value	Mean	SD	MAE	Mean	SD	MAE	mean	SD	MAE	rel. MAE 1	rel. MAE 2
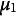	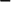 1.000	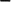 1.038	0.173	0.116	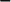 0.825	0.076	0.176	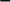 1.031	0.175	0.120	1.520	1.037
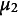	0.000	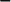 0.015	0.272	0.215	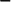 0.040	0.067	0.062	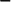 0.020	0.294	0.233	0.287	1.086
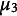	1.000	1.033	0.195	0.143	0.757	0.084	0.243	1.029	0.207	0.153	1.698	1.068
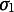	1.000	0.985	0.059	0.042	1.026	0.035	0.035	0.987	0.058	0.042	0.832	0.997
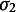	1.000	0.967	0.111	0.082	1.278	0.033	0.278	0.966	0.112	0.083	3.370	1.010
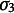	1.000	0.981	0.072	0.049	1.037	0.037	0.043	0.984	0.067	0.048	0.884	0.990
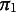	0.800	0.758	0.151	0.110	0.894	0.066	0.100	0.760	0.152	0.109	0.913	0.993
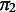	0.100	0.150	0.161	0.123	0.001	0.032	0.101	0.148	0.162	0.122	0.819	0.990
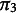	0.100	0.092	0.061	0.050	0.105	0.059	0.047	0.092	0.062	0.051	0.943	1.023
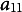	0.750	0.734	0.087	0.056	0.794	0.025	0.045	0.734	0.090	0.059	0.806	1.050
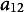	0.125	0.146	0.099	0.073	0.021	0.008	0.104	0.146	0.106	0.075	1.438	1.036
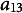	0.125	0.119	0.058	0.047	0.185	0.024	0.060	0.119	0.059	0.048	1.269	1.021
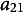	0.125	0.128	0.088	0.063	0.000	0.001	0.125	0.131	0.097	0.069	1.983	1.091
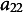	0.750	0.747	0.114	0.083	1.000	0.001	0.250	0.738	0.131	0.092	2.994	1.104
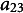	0.125	0.125	0.082	0.062	0.000	0.000	0.125	0.130	0.091	0.067	2.031	1.082
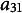	0.125	0.116	0.062	0.048	0.150	0.027	0.030	0.115	0.063	0.049	0.624	1.032
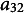	0.125	0.143	0.101	0.076	0.045	0.008	0.080	0.146	0.106	0.081	1.052	1.056
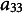	0.750	0.742	0.087	0.062	0.805	0.025	0.056	0.740	0.093	0.068	0.899	1.095
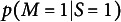	–	–	–	–	0.040	0.015	–	–	–	–	–	–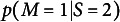	–	–	–	–	0.552	0.024	–	–	–	–	–	–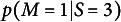	–	–	–	–	0.067	0.022	–	–	–	–	–	–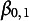	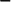 5.000	–	–	–	–	–	–	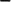 6.149	25.490	1.497	–	–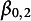	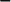 5.000	–	–	–	–	–	–	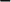 7.153	40.582	2.739	–	–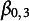	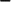 5.000	–	–	–	–	–	–	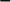 6.472	38.998	1.861	–	–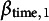	0.125	–	–	–	–	–	–	0.154	0.605	0.039	–	–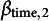	0.125	–	–	–	–	–	–	0.184	1.102	0.075	–	–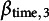	0.125	–	–	–	–	–	–	0.188	1.815	0.074	–	–

*Note*: Values shown are the true value of each parameter, and the mean (mean), standard deviation (SD), and mean absolute error (MAE) of the parameter estimates, for the MAR, MNAR (state), and MNAR (time) model. The value of “rel. MAE 1” is the ratio of the mean absolute error of the MNAR (state) over the MAR model, and the value of “rel. MAE 2” is the ratio of the mean absolute error of the MNAR (time) over the MAR model. Note that the SDs of 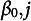 and 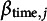 are relatively high. Whilst the estimates are generally accurate, there are rare outlying estimates which inflate these SDs.

This final simulation shows that when modeling patterns of missing data in HMMs, care should be taken in how this is done. An increase in missing data over time could be due to an underlying higher prevalence of states which result in more missing data, and/or a state-independent increase in missingness over time. In applications where the true reason and pattern of missingness is unknown, it is then advisable to start by allowing for both state- and time-dependent missing data, selecting simpler options when this is warranted by the data.

## 4 Application to the National Institute of Mental Health Schizophrenia Collaborative Study

In applying HMMs to the National Institute of Mental Health Schizophrenia Collaborative Study, we assume the severity of schizophrenia is characterized by abrupt – rather than continuous—changes. We fitted HMMs in which we either assumed ratings are MAR, or assume ratings are MNAR and allow missingness to be both state- and time-dependent. For each type of model (MAR or MNAR), we fit versions with 2, 3, 4, or 5 states. Both types of model assume imps79, the IMPS Item 79 ratings, follow a Normal distribution, with a state-dependent mean and SD. No additional covariates were included on these means, as the states are intended to capture all the important determinants of illness severity. To model effects of drug, we allow transitions between states, as well as the initial state, to depend on a dummy-coded covariate drug (1 for medication, 0 for placebo). Whilst the initial measurement at week 0 was made before administering the drug, we allow the initial state at week 0 to depend on drug in order to account for any potential pre-existing differences between the conditions. In the MNAR models, the missingness variable is modeled with a logistic regression, using week (between 0 and 5) and the dummy-coded main variable (1 for main measurement occasion, 0 for the other occasions) as predictors, as these were found to be important predictors in the (state-independent) logistic regression analysis reported earlier (Table 1). All models were estimated by maximum likelihood using the EM algorithm implemented in depmixS4 (Visser & Speekenbrink, 2010). Table 8 contains the goodness-of-fit statistics for all the fitted models.

**Table 8:** Modeling results for the MAR and MNAR HMM with 2-5 latent states

Model	#States	Log likelihood	#par	AIC	BIC
MAR	2	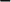 2422.675	16	4865.350	4919.146
	3	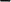 2266.603	30	4577.206	4695.558
	4	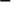 2225.871	48	4527.742	4732.168
	5	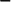 2182.390	70	4480.779	4792.798
MNAR	2	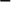 3074.628	22	6181.256	6267.330
	3	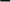 2889.040	39	5840.079	6006.848
	4	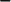 2841.111	60	5782.222	6051.203
	5	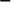 2800.336	85	5746.671	6139.385

*Note*: Note that the likelihood and hence the AIC and BIC values cannot be compared between the MAR and MNAR models, as the latter are based on the additional missingness variable.

**Figure 4:**
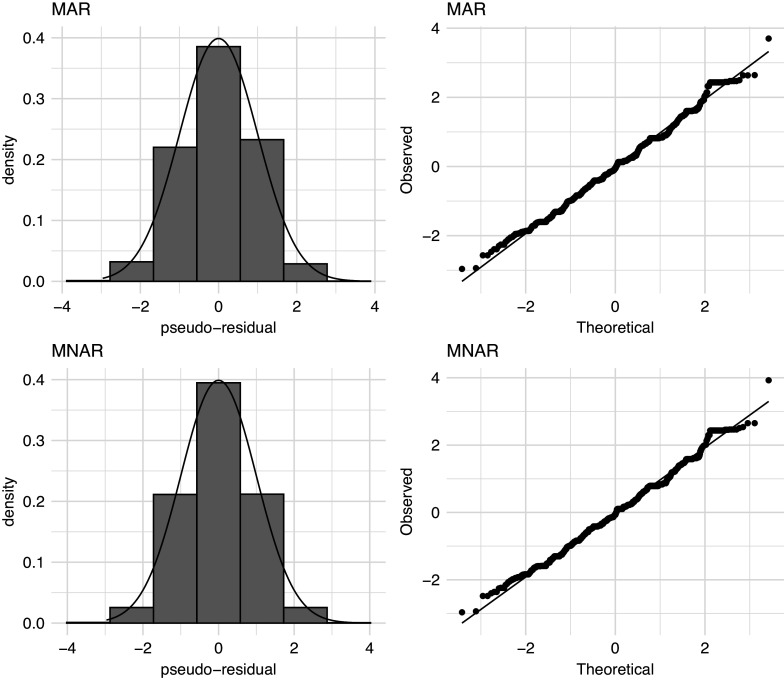
Histograms and QQ plots of the pseudo-residuals for the MAR and MNAR model.

For both the MAR and MNAR models, the BIC indicates a three-state model fits best, whilst the AIC indicates a five-state model (or higher) fits best. Favouring simplicity, we follow the BIC scores here, and focus on the three-state models. Considering the absolute fit to the data, the pseudo-residuals of the three-state MAR and MNAR models (Figure 4) are similar. Whilst there are to-be-expected deviations due to the mostly discrete nature of the ratings, the distribution of the pseudo-residuals is close to standard Normal, indicating a satisfactory fit to the data. As such, there is no reason to doubt the assumption that the IMPS ratings follow a state-conditional Normal distribution.

We first consider the parameter estimates of the MAR model. The estimated means and SD for the severity of symptoms are 

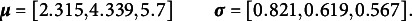

Hence, the states are ordered, with state 1 being the least severe, and state 3 the most severe. The prior probabilities of the states, for treatment with placebo and medication respectively, are 

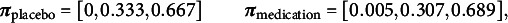

and the transition probability matrices (with initial states in rows and subsequent states in columns) are 

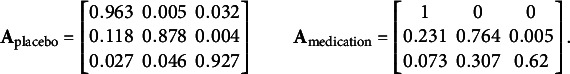

As expected, the initial state probabilities show little difference between the treatments (as the initial measurement was conducted before treatment commenced), but the transition probabilities indicate that for those who received medication, transitions to less severe states are generally more likely, indicating effectiveness of the drugs. This is particularly marked for the most severe state, where the probability of remaining in that state is 0.927 with placebo, but 0.62 with medication. Also note the difference between the transition probabilities for the least severe state: when administered medication, the probability of remaining in the least severe state equals approximately 1, whereas that is not the case for the placebo group.

We next consider the three-state MNAR model. The means and SD for the severity of symptoms are 

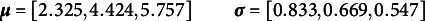

showing the same ordering of states in terms of severity. The prior probabilities for placebo and medication conditions are 

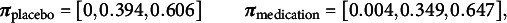

and the transition probability matrices are 

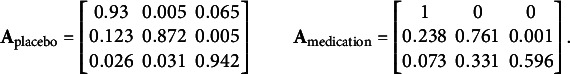

These estimates are close to those of the MAR model, indicating little initial difference between the conditions, but effectiveness of the drugs reflected in the transition probabilities, which are higher toward the less severe states in the medication compared to the placebo condition.

**Figure 5:**
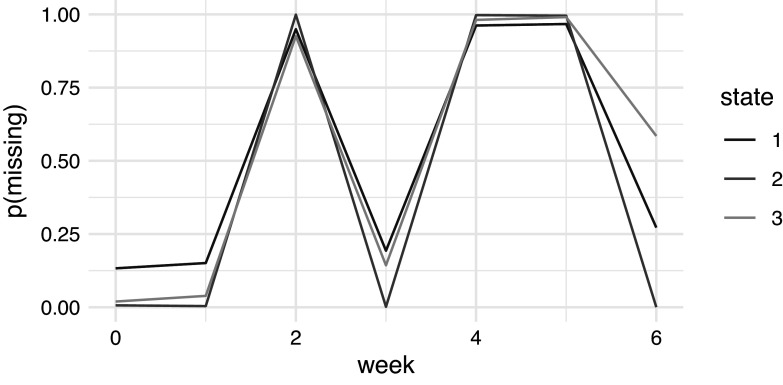
Predicted probability of missing IMPS Item 79 ratings by week for each state in the three-state MNAR hidden Markov model.

Results of the state-dependent models for missingness are provided in Table 9. For all three states, the confidence interval for the effect of main excludes 0, indicating a significantly lower proportion of missing ratings at the main measurement occasions. In state 1 and 3, the confidence interval for the effect of week also excludes 0, indicating a higher rate of missing ratings over time, possibly due to attrition. For state 2, the effect of week is not significant. Figure 5 depicts the predicted probability of missing ratings for each state and week. This shows that in state 2, the chance of missing data on the main measurement occasions is small at 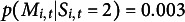 , while it is high at 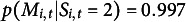 on the other weeks. In the other states, the probabilities are less extreme, with missing (and non-missing) data occurring on both the main measurement weeks as well as the other weeks. In the final week 6, those in the most severe state 3 are the most likely to have missing data with 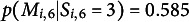 . For those in the least severe state 1, the probability of missingness in week 6 is also substantial at 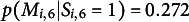 .

**Table 9:** Parameter estimates of the state dependent logistic regression models for missingness, with lower and upper reflecting the lower and upper bounds of the approximate 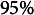 confidence intervals

state	parameter	estimate	lower	upper
1	(Intercept)	2.634	1.996	3.273
	week	0.149	0.027	0.270
	main	−4.511	−5.037	−3.984
2	(Intercept)	7.976	0.971	14.980
	week	−0.510	−2.050	1.030
	main	−13.011	−20.222	−5.800
3	(Intercept)	1.107	0.305	1.909
	week	0.711	0.537	0.884
	main	−5.028	−5.831	−4.225

**Figure 6:**
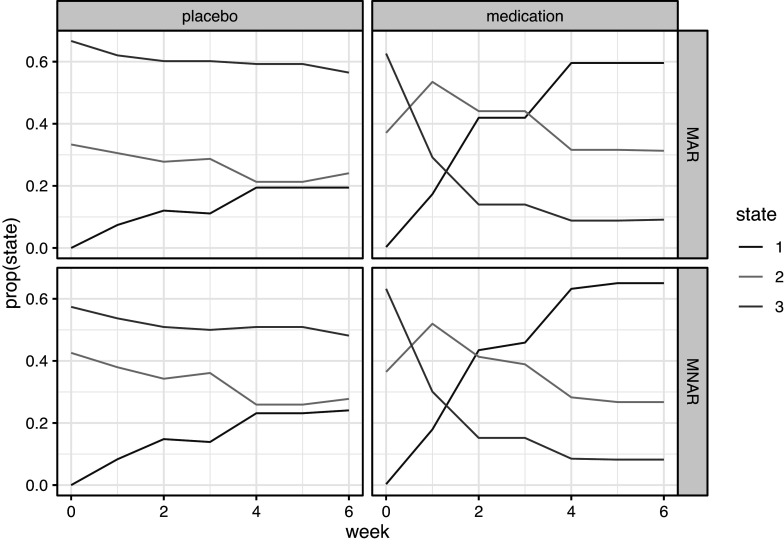
Proportions of MAP state assignments over weeks for the medication and placebo groups, according to the MAR and MNAR model.

The intercepts for the state-dependent missingness model are also worth considering, especially in interaction with the time-dependent effects. The probability of missingness differs between the states, such that the more extreme states have more missingness. The middle state with medium severe symptoms shows a particular missingness pattern where all the main measurements are almost certainly present whereas the non-main measurements are all missing (see also Figure 6). Both in the least and most severe states, week and main have significant effects. The pattern of correlation between missingness and severity is however complex. In the least severe state, missingness is relatively high at the start and increases only minimally during the study’s 6 weeks. In the most severe state, this is very different: early on the probability of missingness is very low but then steeply increases such that by week 6 the probability of missingness is 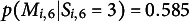 .

Disregarding the modeling of missingness, the parameters of the MAR and MNAR model seem reasonably close. This could be an indication that missingness is independent of the hidden states and data are possibly MAR. As discussed previously, the likelihood of the MAR is not directly comparable to that of the MNAR model, as the latter is defined over two variables (the imps79 rating and the binary missing variable), while the former involves just a single variable (imps79). We therefore compare the MNAR model to a constrained version where the parameters of the missingness model are forced to be identical over the states. Unlike the MAR model, this restricted version of the MNAR model accounts for patterns of missingness, allowing these to depend on week and main, but crucially not on the hidden state. A likelihood ratio test indicates that this restricted model fits significantly less well, 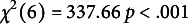 . Hence, there is evidence that the MNAR model is preferable to the MAR model and that missingness is indeed state-dependent.

Whilst the MAR and MNAR model provide roughly equivalent parameters for the severity ratings in the three states, when comparing the MAP state classifications by the Viterbi algorithm (Figure 6), we see that state classifications for the MAR model tend toward the more severe states. According to the MNAR model, during the main measurement occasions, missing values are relatively likely in the least severe state 1. Hence, those with missing values are more likely to be assigned to the least severe state. This is in line with the analysis of Hedeker and Gibbons (1997), who found evidence that dropouts in the medication condition showed more improvement in their symptoms before dropping out than those participants who completed the study.

It is worthwhile to note that the MAP states are also determined for time points with missing data, as the transition probabilities make certain states more probable than others, even when there is no direct measurement available. This provides a potentially meaningful basis to impute missing values with e.g., the state-conditional mean. Another option is to impute with an expected rating computed as a weighted sum of all the state-conditional means, weighted by the posterior probability of the states. As imputation is not the focus of this study, we leave the usefulness of such approaches to be investigated in future work.

## 5 Discussion

Previous work on missing data in HMMs has mostly focussed on cases where missing values are assumed to be MAR. Here, we addressed situations where data is MNAR, and missingness depends on the hidden states. Simulations showed that including a submodel for state-dependent missingness in a HMM is beneficial when missingness is indeed state-dependent, whilst relatively harmless when data is MAR. However, when the form of state-dependent missingness is misspecified (e.g., the effect of measurable covariates on missingness is ignored), results may be biased. In practice, it is therefore advisable to consider the potential effect of covariates in the state-dependent missingness models. A reasonable strategy is to first model patterns of missingness through e.g., logistic regression, and then include important predictors from this analysis into the state-dependent missingness models. Applying this strategy to a real example about severity of schizophrenia in a clinical trial with substantial missing data, we showed that assuming data is MAR may lead to possible misclassification of patients to states (toward more severe states in this example).

The application showed a complex pattern of interaction between severity of symptoms and probability of missingness. For patients with the most severe symptoms the initial probability of missingness is low whereas it steeply increases over the course of the study. This could be the result of two factors: first, patients with severe symptoms have stronger motivation to participate and hence to provide data at the outset of the study. Secondly, when (serious) symptoms persevere throughout the study, the motivation may drop quickly and this is evidenced by a high drop-out rate at the final measurement occasion of 59%. This pattern is different for patients in the least severe symptom state: their initial probability of missing data starts at moderate levels, and slowly increases during the study. It may be that their motivation to participate declines somewhat and drop-out hence increases to 27% toward the end of the study. Here motivation could be interpreted broadly as any circumstance that prevents the patients from providing data for the study. Rather than merely internal, motivational factors, these could also be illness related factors that prevent the patient from providing data. These results provide interesting directions for future studies on the intricate relationship between patient factors, missing data and treatment effectiveness. Interestingly, the group of patients with medium severity of symptoms has the least drop-out and missingness throughout the study. This group apparently has a strong motivation to participate; they could expect to gain much from treatment, whilst their symptoms are not so severe that they are prevented from participating in the measurements. Importantly, these types of patterns of interaction between missingness and severity are only revealed by studying these data using HMMs rather than linear models.

Whilst subtle, the MAR and MNAR models showed interesting and potentially clinically meaningful differences. Although the ground truth is unavailable in such real applications, model comparison can be used to justify a state-dependent missingness model. Using flexible analysis tools such as the depmixS4 package (Visser & Speekenbrink, 2010) makes specifying, estimating, and comparing HMM with missing data specifications straightforward. And, as was shown in the simulations studies, even if data is MAR, the MNAR model performs as well as the MAR model. There is then little reason to ignore potentially non-ignorable patterns of missing data in hidden Markov modeling.

Recently, Pandolfi et al. (2023) proposed a different method to deal with MNAR data in HMMs.^4^ They developed a HMM for multivariate Normal data, where intermittent missing data is assumed MAR, whilst allowing missing data due to dropout to depend on the hidden state at the previous time point via an observed and absorbing “dropout state”. In applications where it is important to distinguish between intermittent missingness and dropout, it could be of interest to combine their method with ours, allowing intermittent missingness to be MNAR and state-dependent via a state-dependent missingness model (as done here), and including an absorbing dropout state to distinguish MNAR dropout from intermittent MNAR data.

Another approach to dealing with non-ignorable missingness (MNAR) is the pattern-mixture approach of Little (1993, 1994). The main idea of this approach is to group units of observations (e.g., patients) by the pattern of missing data, and allowing the parameters of a statistical model for the observations 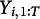 to dependent on the missingness *pattern* 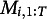 . There are certain similarities between this approach and modeling missingness as state-dependent. Rather than conditionalizing on a pattern of missing values, a hidden Markov model conditionalizes on a pattern (sequence) of hidden states, 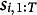 , and the marginal distribution of the observations is effectively a multivariate mixture (25)

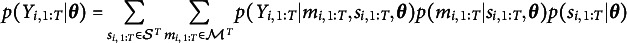

(note that 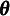 here includes all parameters, so also 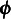 ). A pattern-mixture model would instead propose (26)

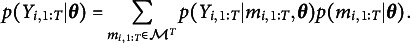

Trivially, if we set the number of hidden states to 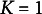 , both models are the same. Another trivial equivalence is via a one-to-one mapping between 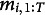 and 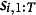 , by e.g., setting 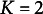 , assuming the Markov process is of order *T*, and fixing 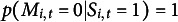 and 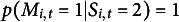 . More interesting is to investigate cases where the procedures are similar, but not necessarily equivalent. The general pattern-mixture model is often underidentified (Little, 1993). For univariate time-series of length *T*, there are 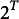 possible missing data patterns. Without further restrictions, estimating the mean and covariance matrices separately for each pattern of missing data is not possible, due to the structural missing data in those patterns. The state-dependent MNAR hidden Markov model is identifiable insofar as the HMM for the observed variable *Y* is identifiable. It is convenient, but not necessary, to assume a first-order Markov process. Higher-order Markov processes may allow the model to capture complex patterns of missingness. Another option is to use the missingness indicator 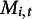 as a covariate on initial and transition probabilities, rather than a dependent variable. We leave investigation of such alternative models to future work.

## Footnotes

1
https://hedeker.people.uic.edu/SCHIZREP.DAT.txt.

2 The dataset provided contains some non-integer values for these ratings, presumably given to provide a finer-grained evaluation by the clinician.

3 All code for the simulations, and the analysis of the application, is available at https://osf.io/7td32/.

4 We were made aware of this paper, which appeared after the research reported here was completed, by an anonymous reviewer.

## Appendix

**Table A1:** Results of Simulation 5 (MNAR, low variance)

		MAR			MNAR			
Parameter	True value	Mean	SD	MAE	mean	SD	MAE	rel. MAE
	1.000	1.129	0.332	0.247	1.029	0.286	0.197	0.797
	0.000	0.132	0.388	0.332	0.010	0.378	0.308	0.928
	1.000	0.976	0.423	0.320	1.073	0.447	0.326	1.020
	1.000	0.953	0.120	0.087	0.979	0.114	0.072	0.833
	1.000	0.939	0.209	0.167	0.957	0.212	0.160	0.958
	1.000	0.972	0.138	0.100	0.946	0.151	0.111	1.114
	0.333	0.355	0.220	0.185	0.324	0.171	0.138	0.747
	0.333	0.376	0.273	0.230	0.361	0.252	0.209	0.910
	0.333	0.269	0.189	0.166	0.315	0.193	0.160	0.969
	0.500	0.524	0.189	0.152	0.517	0.144	0.110	0.728
	0.250	0.268	0.199	0.159	0.252	0.180	0.145	0.914
	0.250	0.208	0.156	0.130	0.231	0.141	0.115	0.882
	0.250	0.263	0.197	0.157	0.225	0.156	0.124	0.793
	0.500	0.526	0.244	0.202	0.530	0.213	0.172	0.856
	0.250	0.211	0.182	0.149	0.244	0.178	0.143	0.957
	0.250	0.270	0.187	0.148	0.242	0.149	0.118	0.797
	0.250	0.277	0.220	0.178	0.259	0.195	0.157	0.880
	0.500	0.453	0.184	0.148	0.499	0.165	0.128	0.861
	0.050	–	–	–	0.071	0.098	0.059	–
	0.250	–	–	–	0.257	0.154	0.122	–
	0.500	–	–	–	0.492	0.138	0.098	–

*Note*: Values shown are the true value of each parameter, and the mean (mean), standard deviation (SD), and mean absolute error (MAE) of the parameter estimates, for both the MAR and MNAR model. The value of “rel. MAE” is the ratio of the mean absolute error of the MNAR over the MAR model.

**Table A2:** Results of Simulation 6 (MAR, low variance)

		MAR			MNAR			
Parameter	True value	Mean	SD	MAE	Mean	SD	MAE	rel. MAE
	1.000	1.090	0.388	0.278	1.079	0.397	0.277	0.996
	0.000	0.017	0.397	0.323	0.026	0.397	0.322	0.997
	1.000	1.079	0.378	0.272	1.052	0.361	0.257	0.945
	1.000	0.956	0.124	0.091	0.959	0.134	0.094	1.035
	1.000	0.938	0.212	0.167	0.948	0.213	0.164	0.983
	1.000	0.955	0.124	0.091	0.961	0.123	0.087	0.955
	0.333	0.312	0.203	0.169	0.318	0.199	0.163	0.965
	0.333	0.368	0.273	0.229	0.356	0.263	0.220	0.957
	0.333	0.320	0.196	0.162	0.326	0.195	0.161	0.991
	0.500	0.489	0.181	0.143	0.496	0.170	0.133	0.933
	0.250	0.270	0.203	0.161	0.262	0.191	0.153	0.950
	0.250	0.240	0.164	0.131	0.243	0.163	0.129	0.989
	0.250	0.229	0.177	0.143	0.226	0.168	0.135	0.941
	0.500	0.535	0.228	0.185	0.535	0.219	0.178	0.959
	0.250	0.236	0.181	0.142	0.238	0.178	0.140	0.985
	0.250	0.234	0.166	0.134	0.236	0.162	0.129	0.963
	0.250	0.271	0.205	0.166	0.261	0.195	0.159	0.956
	0.500	0.495	0.176	0.137	0.503	0.168	0.134	0.974
	0.250	–	–	–	0.254	0.129	0.086	–
	0.250	–	–	–	0.248	0.132	0.091	–
	0.250	–	–	–	0.251	0.119	0.079	–

*Note*: Values shown are the true value of each parameter, and the mean (mean), standard deviation (SD), and mean absolute error (MAE) of the parameter estimates, for both the MAR and MNAR model. The value of “rel. MAE” is the ratio of the mean absolute error of the MNAR over the MAR model.

**Table A3:** Results of Simulation 7 (MNAR, low variance)

		MAR			MNAR			
Parameter	True value	Mean	SD	MAE	Mean	SD	MAE	rel. MAE
	1.000	0.983	0.090	0.072	1.004	0.072	0.056	0.782
	0.000	0.038	0.134	0.112	0.001	0.094	0.074	0.659
	1.000	1.069	0.124	0.107	1.012	0.083	0.065	0.607
	1.000	1.004	0.040	0.031	0.999	0.035	0.028	0.896
	1.000	0.989	0.046	0.035	0.994	0.035	0.028	0.802
	1.000	0.981	0.044	0.037	0.996	0.034	0.028	0.750
	0.800	0.858	0.087	0.090	0.794	0.077	0.061	0.677
	0.100	0.086	0.094	0.079	0.109	0.092	0.077	0.965
	0.100	0.055	0.043	0.053	0.097	0.051	0.041	0.768
	0.800	0.816	0.034	0.030	0.798	0.029	0.022	0.741
	0.150	0.148	0.047	0.037	0.153	0.043	0.034	0.922
	0.050	0.036	0.028	0.026	0.048	0.027	0.022	0.853
	0.038	0.043	0.023	0.018	0.038	0.018	0.014	0.788
	0.850	0.862	0.042	0.035	0.849	0.032	0.025	0.711
	0.112	0.095	0.035	0.032	0.113	0.027	0.021	0.651
	0.025	0.032	0.020	0.017	0.025	0.011	0.009	0.557
	0.075	0.098	0.050	0.039	0.078	0.025	0.020	0.510
	0.900	0.870	0.046	0.037	0.897	0.021	0.017	0.445
	0.050	–	–	–	0.050	0.016	0.013	–
	0.250	–	–	–	0.251	0.030	0.023	–
	0.500	–	–	–	0.501	0.019	0.015	–

*Note*: Values shown are the true value of each parameter, and the mean (mean), standard deviation (SD), and mean absolute error (MAE) of the parameter estimates, for both the MAR and MNAR model. The value of “rel. MAE” is the ratio of the mean absolute error of the MNAR over the MAR model.

**Table A4:** Results of Simulation 8 (MAR, low variance)

		MAR			MNAR			
Parameter	True value	Mean	SD	MAE	Mean	SD	MAE	rel. MAE
	1.000	1.015	0.119	0.091	1.015	0.121	0.091	1.002
	0.000	0.005	0.133	0.104	0.005	0.135	0.106	1.013
	1.000	1.006	0.073	0.057	1.006	0.073	0.057	1.001
	1.000	0.995	0.051	0.039	0.995	0.052	0.039	1.003
	1.000	0.989	0.049	0.038	0.988	0.049	0.038	1.004
	1.000	0.998	0.029	0.022	0.998	0.028	0.022	0.996
	0.800	0.784	0.110	0.086	0.783	0.111	0.086	1.000
	0.100	0.122	0.124	0.100	0.124	0.125	0.100	1.003
	0.100	0.094	0.055	0.044	0.093	0.055	0.044	1.002
	0.800	0.796	0.042	0.032	0.796	0.042	0.032	0.993
	0.150	0.156	0.059	0.046	0.156	0.059	0.046	0.991
	0.050	0.048	0.033	0.027	0.048	0.033	0.027	0.994
	0.038	0.038	0.025	0.019	0.038	0.025	0.020	1.010
	0.850	0.849	0.043	0.033	0.848	0.044	0.034	1.019
	0.112	0.114	0.035	0.026	0.114	0.036	0.026	1.030
	0.025	0.025	0.015	0.012	0.025	0.015	0.012	1.005
	0.075	0.078	0.034	0.026	0.078	0.034	0.025	0.993
	0.900	0.897	0.028	0.021	0.897	0.028	0.021	1.001
	0.250	–	–	–	0.250	0.024	0.019	–
	0.250	–	–	–	0.249	0.021	0.016	–
	0.250	–	–	–	0.250	0.015	0.012	–

*Note*: Values shown are the true value of each parameter, and the mean (mean), standard deviation (SD), and mean absolute error (MAE) of the parameter estimates, for both the MAR and MNAR model. The value of “‘rel. MAE” is the ratio of the mean absolute error of the MNAR over the MAR model.
